# Eggs, ethics and exploitation? Investigating women's experiences of an egg sharing scheme

**DOI:** 10.1111/j.1467-9566.2012.01467.x

**Published:** 2012-03-23

**Authors:** Erica Haimes, Ken Taylor, Ilke Turkmendag

**Affiliations:** Policy, Ethics and Life Sciences (PEALS) Research Centre, Newcastle University

**Keywords:** exploitation, egg sharing, stem cells, commodification, IVF

## Abstract

There is a growing global demand for human eggs for the treatment of sub-fertile women and for stem cell-related research. This demand provokes concerns for the women providing the eggs, including their possible exploitation, whether they should be paid, whether they can give properly informed consent and whether their eggs and bodies are becoming commodified. However, few of the debates have benefitted from insights from the women themselves. We address this gap in knowledge by reporting on a study investigating women’s views and experiences of a scheme in which they can volunteer, in their capacity as fertility patients, to ‘share’ their eggs with researchers and receive a reduction in *in vitro* fertilisation fees. We focus our discussion on the question of exploitation, a concept central to many sociological and ethical interests. In brief, our analysis suggests that while interviewees acknowledge the potential of this scheme to be exploitative, they argue that this is not the case, emphasising their ability to act autonomously in deciding to volunteer. Nonetheless, these freely made decisions do not necessarily take place under circumstances of their choosing. We discuss the implications of this for egg provision in general and for understandings of exploitation.

## Introduction

The growing global demand for human eggs, for the treatment of sub-fertile women and for research, has been accompanied by concerns for the women providing the eggs (Haimes and Taylor 2011): are they being exploited; should they be paid; can they give properly informed consent and are their eggs and bodies being commodified by the ‘tissue economy’ (Waldby and Mitchell 2006) in which they play such a central role? These and related issues have been debated recently in the UK by two prominent organisations, the Nuffield Council on Bioethics (NCoB 2011) and the Human Fertilisation and Embryology Authority (HFEA 2011).

Few debates on the ethics of egg provision and procurement have benefitted from insights from the women themselves. We address this gap in knowledge by reporting on women’s views and experiences of one particular scheme in which they can volunteer, in their capacity as fertility patients, to ‘share’^1^ their eggs with researchers, receiving a reduction in *in vitro* fertilisation (IVF) fees in return. The Newcastle egg sharing for research scheme (NESR) was set up to acquire eggs as part of a research collaboration between the local fertility clinic and stem cell research institute. It was established within a developing international landscape of debates on the moral, economic and gender aspects of acquiring and using human eggs for stem cell research. Those debates both shaped and have been shaped by the NESR.

While all the concerns raised require detailed consideration, we focus on the question of exploitation, a concept central to many sociological and ethical discussions. A conceptual and empirical exploration of exploitation in egg provision contributes to several related substantive and epistemological topics, such as the characteristics of the IVF–stem cell interface (Franklin 2006), the socio-ethical questions raised by the use of human reproductive tissue in research, socioeconomic practices of the provision, acquisition and brokering of human tissue (including the relationships between tissue providers, clinical brokers and end user scientists (Haimes and Taylor 2011, NCoB 2011), the nature of participation in clinical research and, last but not least, the relationship between sociology and ethics (Haimes and Williams 2007). Illustrating how exploitation is asserted, positioned, debated and challenged in one forum furthers understanding of the concept itself and of other areas of social life in which it features as a major concern.

## Women, eggs and exploitation?

Women have provided^2^ eggs to help other women get pregnant since the early 1990s, a practice often labelled ‘egg sharing’ (Ahuja *et al.* 1999). In the 2000s the use of human eggs in research developed from investigations into reproduction and fertility to include stem cell related techniques such as somatic cell nuclear transfer (SCNT). Also known as therapeutic cloning, SCNT involves removing the nucleus from an egg, replacing it with the nucleus of a somatic cell (a non-reproductive cell, such as a skin cell) then stimulating the egg to develop into an embryo from which embryonic stem cell lines can be derived. The intention is to use those cell lines in the development of patient-specific and disease-specific therapies. In the UK the imaginary of the therapeutic promise proved compelling (Donaldson Report 2000) and SCNT was approved through an amendment of the Human Fertilisation and Embryology Act in 2002. Little attention was paid to who provided the eggs or the circumstances under which they were obtained, leading Dickenson to suggest that ‘the lady vanished’ (2006).^3^

### The NESR

In May 2005 researchers at Newcastle University, UK, became the first team to create a human blastocyst (a very early embryo of approximately 150 cells) using nuclear transfer techniques. They had used eggs that had failed to fertilise during IVF and argued that ‘if the promise of this new science is to achieve its potential’ (Stojkovic *et al.* 2005: 226) ‘fresh eggs’, obtained before fertilisation had been attempted, were needed. This would mean that the women providing the eggs would have no knowledge of the potential of those eggs to achieve their own goals of a pregnancy before they went to research.

The Newcastle team requested approval from the HFEA in May 2005 to ask IVF women who were predicted to produce 12 or more eggs to donate two fresh eggs to SCNT research, on the grounds that statistically this would not affect their pregnancy chances. However, women proved reluctant to provide eggs. In a previous study we, coincidentally, interviewed women who had been approached under this 12+ scheme, who explained they could not contemplate giving eggs away before knowing if they were going to be of any use in their own attempts to have a baby. Since these were women who had provided embryos for research, this indicates three important points: firstly, they judge the value of eggs and embryos in terms of their usefulness in producing a baby rather than giving them any inherent moral status; secondly, women can exercise autonomy and resist helping research, if they deem it to be against their interests; and thirdly, that an understanding of what lies behind decisions to provide tissue for research benefits greatly from gaining the perspectives of those asked to provide that tissue (Haimes *et al.* 2008).

Since this scheme produced too few eggs to progress their work the researchers applied to the HFEA in 2006 for a licence for a scheme in which IVF women could be given a fee discount of £1500 for supplying half their fresh eggs retrieved during a treatment cycle. They argued that this mirrored the well-established ‘egg sharing’ for treatment (EST) scheme, in which recipients paid for the IVF treatment of the woman providing the eggs in return for half her eggs. This positioning of the new egg sharing for research (ESR) scheme alongside EST was seen by Roberts and Throsby (2008: 161) as a way of indicating that ESR was simply business as usual, thus minimising any suggestion that it constitutes a significantly different practice.

Following a public consultation (HFEA 2006) the NESR was licensed in February 2007 and funding for the SCNT research and the £1500 discount was secured from the UK Medical Research Council in September 2007. The arrangements for the licensed scheme were: (i) women had to volunteer for the NESR rather than be asked directly to provide eggs; (ii) women should have had IVF previously to confirm this was required and to indicate the likely number of eggs they would produce; (iii) women should be under 35; (iv) information about the scheme had to be conveyed and the consenting process conducted by an independent research nurse; (v) if women produced six or more eggs, they would keep half and half would go to research, allocated one-by-one, immediately on retrieval, with no regard to quality; if they produced five or fewer eggs they would keep them all and still receive the discount; and (vi) the woman could change her mind at any time up to egg retrieval but would then have to pay the full cost of treatment.

To understand how this scheme was received, it is necessary to grasp how the social practices of egg provision and procurement in general, and for stem cell research, have been debated.

### Egg provision in general

The growth in demand for eggs has been accompanied by literature debating the practices emerging to satisfy that demand. The literature covers a range of countries, categories of women acting as providers (IVF or non-IVF), the purposes of provision (treatment or research), the circumstances of provision and disciplinary perspectives. Five areas repeatedly cause concern: (i) the validity of SCNT research; (ii) the possible commodification of human eggs and bodies; (iii) the effectiveness of the informed consent; (iv) the role of undue inducement; and (v) whether women were being exploited in this drive to increase the supply of eggs (for example, Braude *et al.* 2005, Baylis 2009, Baylis and McLeod 2007, Dickenson 2002, Gupta 2011).

The literature displays varying degrees of conceptual precision and analytical depth: ‘inducement’, ‘consent’, ‘exploitation’ are often conflated and debates about payment are often unclear on what constitutes payment, let alone whether the payment is deemed to be a return for the eggs or for the woman’s labour or for the risks undertaken. This is less a criticism of the literature than an acknowledgement of the extensive concerns provoked by such practices and by the speed with which they have developed. The complexity of these discussions is testimony to the complexity of the practices of acquiring, providing and brokering eggs for research and to the need for continued scrutiny (Haimes and Taylor 2011).

### Egg provision for stem cell research

The NESR has attracted a range of criticisms and similar schemes elsewhere are rare. Criticisms include the view that women are being ‘paid to share’ (Roberts and Throsby 2008) and that the reduction in fees constitutes an inducement, exploiting the vulnerable, desperate and poor and compromising the patient’s capacity to give her informed consent. It is argued that more widely available National Health Service (NHS)-funded IVF would mean that egg sharing would not exist and that which might be termed truly altruistic donations would be possible (Ashcroft 2003). Interestingly, the Newcastle Fertility Clinic has been successful in ensuring that national guidelines on access to three NHS-funded IVF cycles have been implemented by local primary care trusts; only 27% of trusts in the UK have done so (All Party Parliamentary Group on Infertility 2011). Nonetheless, concern over exploitation has been expressed nationally and internationally. For example, the Singapore Bioethics Advisory Committee said specifically about the NESR, that it was ‘seriously concerned with the possibility of exploitation of women, especially those of limited economic means’ (Singapore Bioethics Advisory Committee 2008: para 4.23). On the other hand, the European Society of Human Reproduction and Embryology Task Force on Ethics and Law concluded that egg sharing is acceptable within certain protective structures (Pennings *et al.* 2007).

Waldby found that in India and China the combination of impoverished women, established fertility clinics and EST ‘set the scene for exploitative forms of oocyte procurement’ for emerging stem cell industries (Waldby 2008: 27), suggesting that EST is not necessarily a benign precedent for ESR. Indeed, in 1998 the HFEA considered banning EST because of concerns about payment (Ahuja *et al.* 1999). The now emblematic Hwang scandal, in which SCNT results were falsified, also raised concerns about egg provision (Tsuge and Hong 2011). Baylis showed that nearly 75% of women providing eggs to Hwang’s research had received cash or in-kind payments and argued that ‘the purchase of eggs for stem cell research raises the spectre of coercion, undue inducement, commodification of reproductive labour and tissues, and harmful exploitation’ (Baylis 2009: 391). Commentators fear that a similar abuse of women could spread in countries that lack regulation and have high poverty levels (Gupta 2011). However, efforts to avoid undue inducement risk ‘the global exploitation’ of economically disadvantaged women (Baylis and McLeod 2007: 726). This neatly sums up the debate between those who favour payment and those who oppose it, depending on which they regard as being the most or least exploitative.

In brief, exploitation is seen to lie, variously, in the actual or possible economic position of providers, the likely vulnerability of providers, payments or lack of payments, the lack of regulation; inappropriate regulation, the interface between fertility clinics and stem cell scientists, the economic inequality between providers and users of eggs, within and between countries and hemispheres and the failure to acknowledge women’s reproductive labour in producing eggs. This observation is not intended to disparage the writers or the seriousness of the circumstances in which some women might find themselves as a result of being involved in egg provision. It simply demonstrates the amount of work that the concept of exploitation has to do.

If we can identify more precisely the circumstances in which women contemplate the provision of eggs (and other human reproductive tissue) and the ‘expertise, experience and interests’ (Waldby 2008: 29) they bring to and develop out of those circumstances, we can identify when, where, by whom and why exploitation occurs, and begin to develop ways of learning from women what resources and skills are needed to diminish it wherever possible. We can start by looking at NESR to see what volunteers say about exploitation.

## Women’s views on exploitation and the NESR

This study aimed to investigate the experiences of women volunteering for the NESR, since it is important to include the perspective of egg providers if we are to understand fully the social and ethical impact of SCNT research. Our central question was ‘does egg “sharing” for SCNT research, in exchange for reduced IVF fees, entail social and ethical costs for those coming forward to participate in the scheme?’ The project was approved by the local research ethics committee. It is important to record that we are employed at the same university as that to which some of those establishing the NESR are affiliated, though it is noteworthy that we were awarded the grant after highly rated external reviews. We had also conducted a study of IVF couples who had provided embryos for stem cell research and have an extensive track record of collaboration with clinical and scientific colleagues on a number of socio-ethical projects. Nonetheless, we recruited a project advisory group to assist in maintaining analytical objectivity. This included a senior scientist with a public record of strongly opposing the NESR and SCNT research using human eggs.

This was a prospective, interview-based, qualitative study designed to gain an understanding of the perspectives and reasoning of volunteers. We used heterogeneity sampling and aimed to recruit sufficient numbers to reach thematic saturation. We interviewed 29 volunteers, 21 comparators (who provided eggs for the treatment of others) and 17 staff. All interviews, except for 10 clinic staff, were conducted by the lead author, enabling ongoing analysis and progressive focusing; all were fully transcribed. We conducted a hermeneutic analysis of transcripts, using constant comparison and category-building procedures followed by category mapping (Silverman 2001). For reasons of space, we focus on the volunteers’ views.^4^

In all 19 analytic themes were identified, of which five were most prominent: the interviewee’s fertility story, money, the NESR, the interviewee’s egg sharing story, and the juggling of considerations in deciding whether to provide eggs. Central to all these is the wish for a baby. Interestingly, given its prominence in the literature, exploitation was not a theme raised explicitly or spontaneously by any interviewees. However, no one expressed surprise at being asked about exploitation, suggesting that they could see its relevance. The interviewer was careful to introduce the topic of exploitation within a discussion of external comments about the scheme, rather than to ask, bluntly, if interviewees themselves were, or felt they were, exploited.

The interviewees’ views on exploitation fell into two broad categories: those who acknowledged the possibility of exploitation, while not agreeing that it occurred, and those who refuted it outright. There were nuanced reflections as interviewees thought out loud about the issue, but no one expressed anything close to a full-hearted agreement that the NESR was exploitative.

### Conceding the possibility of exploitation

There were two broad ways in which the volunteers conceded that the NESR could be exploitative: firstly, in relation to potentially vulnerable groups and secondly, by reference to other situations that were considered to be more exploitative (the comparative language indicating that the NESR had the potential to be exploitative).

#### Vulnerability

Interviewees regarded other volunteers as potentially vulnerable, emotionally and financially. One woman egg ‘sharer’ said ‘I never felt like that’ (M01: 850–903)^5^ though she and her husband commented, ‘that would be the only objection I could think of ... exploiting people who otherwise wouldn’t be able to afford to do it’ (M01/M01P: 1079–92) They later added:

regarding exploiting people and money ... entrance criteria should not just be based on the medical side, it needs to weigh up desperation ... it has to be people’s emotional ability to cope ... there’s financial vulnerability and emotional vulnerability. (M01/M01P: 1979–2014; 2082–2106).

Another couple thought the scheme was ‘a good idea ... It’s different if somebody’s onto their third attempt and they’re a bit more vulnerable’ (M09: 515–38). However, they decided not to continue with NESR, indicating that individuals could resist it.

Another couple, who also withdrew, differed slightly: the woman said, ‘I don’t think it is [exploitative] personally, because I think you can make that decision for yourself as to whether you really want to do it’ but her husband was more hesitant:

If another scheme did it in a different way you could say, ‘That’s more exploitative than the other one’ ... I don’t know if it’s exploitative. I suppose it could be ... I still think you’re paying a heavy price by giving half your chances [away]. (M10: 1713–1756).

Volunteers who withdrew from the NESR are the most likely to be critical of the scheme, but even this (albeit small) group of interviewees never expressed outright concern with exploitation, attributing their decisions to withdraw to personal circumstances.

#### Other situations are more exploitative

A slightly different aspect was suggested by those who compared the NESR with other situations that could be more clearly exploitative. Many interviewees placed the NESR alongside the costs of private fertility treatment. For example:

I can see the argument [about exploitation] ... you hear about women spending thousands of pounds on treatment, 50, 60 thousand pounds ... their life savings, they’ve remortgaged their home ... If you are quite happy to help somebody else to get pregnant or if you are happy enough for your eggs to be researched, I think that’s fine. (M16: 1582–1617)

The NESR was seen as providing a solution to the perceived financial exploitation by private clinics.

For some, providing eggs for research was preferable and less exploitative than providing eggs for the treatment of others. For example:

[S]haring for treatment for other people ... could be classed as more exploitative because of what the consequences are, the emotional things. [The NESR] is a lot easier to get your head around, even though [EST] is free to you. (M01: 850–903)

Another woman described herself as having ‘baby madness’ and would have done anything:

At the time, it was all, ‘that’s good because I’ll be helping someone’ but thinking about it now, [EST] is quite exploitative really ... the fact that I was going to have a child that was half mine out there in the world ... I think maybe they are preying on people but I was probably quite happy to be preyed on if it meant that I was going to get the treatment. (M17: 740–66)

She expresses much of the complexity of the relationship between exploitation and egg sharing: alongside the strong language of being ‘preyed on’ she argues that there is a choice in going along with that, if the benefit is perceived to outweigh the currently perceived consequences. Clearly this points to the need to ensure that couples contemplating either ESR or EST take time and perhaps receive help in thinking through the longer term consequences. (This woman had not received the standard support that the clinic offers as she made only limited inquiries before conceiving naturally.) It also suggests that individuals can ‘choose to be exploited’ if, from their perspective, the benefits outweigh the costs. However, in EST, unlike ESR, the involvement of others, particularly a child, suggests that the choice is not just that of the individual to make.

Egg selling over the Internet was considered as more exploitative than a scheme based in familiar surroundings that was providing treatment in return. As one woman said:

[I]f it was go on the Internet and sell them or go to somewhere where you’re going to receive some treatment, it’s all in one process and you get some benefit from it, for me that’s a much better idea ... [Internet selling is] open to exploitation ... how do you know how many they are taking, are you going to sit in a lab and count them together to cash up your money? (M28: 1248–66)

Another thought the potential for exploitation lay in women selling eggs for money:

I think it’s [NESR] incredibly valuable. I think it’s more important than anyone else will know ... but they should view who they give it to ... if somebody’s saying, ‘I think you should be paying me for my eggs’, that to me is a different category of person. You are exploiting somebody if it’s all about money. (M29: 1328–52)

To sum up the data so far, there is evidence that some of the interviewees conceded the possibility that NESR has the potential to be exploitative but this possibility was not considered to be sufficiently strong to put them off volunteering as sharers.

### Refuting exploitation

The weight of interviewee opinion lay with those who refuted the charge of exploitation, usually on one of four grounds.

#### ‘Women can make their own decisions’

Those who had provided eggs were prominent here. One woman asserted, ‘I wouldn’t say I was exploited at all,’ given that she was the one who approached the clinic, rather than vice versa, ‘No, not at all ... because you weren’t pressurised at all ... we actually brought it up.’ (M02: 904–31). Another sharer returned to the subject:

[J]ust touching upon the ... concerns about exploitation of women ... I don’t think it would actually be called exploitation. Everybody has a choice. You’re not forcing somebody into a corner ... So I don’t think it’s exploitation. (M03: 1655–65)

This was important to others:

I think it’s not exploitation at all ... it’s helping people who otherwise wouldn’t be able to afford the IVF. If somebody felt strongly against it they wouldn’t do it, it’s not like somebody’s got their arm twisted up their back, forcing them into it, it’s their decision ... we phoned the hospital. It’s our decision. (M04: 641–66)

This is echoed by a volunteer who decided not to go ahead:

I don’t believe it at all personally because it was full choice ... it was just a leaflet I had seen, it wasn’t pushed or anything, it was just informative ... If someone’s desperate and they can’t afford a full cycle they may decide to do that, when they don’t really want to, but that’s still their choice. The centre’s not forcing them. (M11: 795–818)

This response is particularly telling as this woman had been accepted for the scheme after multiple cycles of IVF so she had a good sense of the desperation and the financial and emotional costs involved, but still withdrew.

Another woman spelt out the intermingled reasons for denying that the NESR is exploitative:

Everybody’s got their reasons for doing what they do ... it’s your chance to maybe have another child, whether you’re exploited or not. But then research needs to happen as well and there needs to be some plan for getting that done ... No, I don’t think I would feel exploited, no. I think you’ve got your own selfish reasons for doing things so it’s up to you. I know desperation drives you but [pause] you don’t have to do it at the end of the day. (M21: 1128–54)

Proper information was seen as another important counter to the possibility of exploitation. One couple stressed, ‘you go into it more seriously than any couple meeting up, having a one night stand and getting pregnant’ (M15: 1498–1507). Another said:

I think it would be up to the person, as long as they’re getting all the information and people saying to them ‘you’re going to be reducing your chances, you sharing your eggs’. As long as they are fully aware of that, I think the choice would be up to them. (M18: 977–1029)

#### ‘NESR offers another chance’

For some the NESR meant they had a realistic chance of affording an extra (or even just one) IVF cycle.

[The NESR] offers people another hope, rather than exploiting them ... I wouldn’t feel exploited but I can understand how some people may think, ‘ financially I’ll never have the chance to go privately ... I’m only doing it because it’s the only hope’ ... I wouldn’t feel exploited I don’t think. (M23: 966–97)

Another argued:

I think women who ... are in the position that I am are grateful for whatever help they can get ... No, I don’t think it is [exploitative] ... I really, really don’t feel that I would be exploited in any way, shape or form. The fact there’s a facility there that both parties can benefit from, that’s got to be a good thing and it gives light at the end of the tunnel to women like me, thank God. (M27:1755–1807)

#### ‘There should be more treatment available on the NHS’

The topic of IVF funding featured heavily in all interviews. Even interviewees who had access to sufficient finances raised NHS funding, for example, as part of how they juggled whether to have private, NHS, EST or NESR cycles and in which order. For others, the paucity of NHS funding, coupled with the restrictions on access (especially those that stipulated a couple must not already have a child in their relationship) caused a great deal of upset. One interviewee who lived outside the north-east region said:

Nobody’s getting exploited, if they didn’t want to do it they wouldn’t do it, but some people are so desperate for a family. Four and half thousand pounds is a lot of money ... you’ve got to give people a chance ... the NHS waiting list is five years and they’re on about putting it to seven. (M29: 872–918)

A frequent comment was that critics of the NESR should address this issue instead:

[T]he people that are lobbying to say that it’s exploiting people [should] set up another NHS scheme so that people don’t have to wait three years to get NHS treatment ... if you don’t want them to donate their eggs, set up some other scheme where people can get funding, other than having to wait on the NHS ... Do something that’s important that’s going to help people. (M03: 1585–1618)

#### Helping medical research

One characterisation of the ESR scheme is that it produces a ‘win-win’ situation in which patients get cheaper treatment and researchers get eggs. While that needs further deconstruction since the ‘wins’, let alone the costs and the risks, might not be equal for each side, some interviewees thought that helping medical research was itself a counter to the charge of exploitation:

If you’re lucky enough to produce enough eggs and it allows you to have your treatment, but it also helps on the other side, I just think it’s a win-win. (M19: 717–28)

While some commentators have concerns about IVF women being targeted in this way (Ballantyne and de Lacey 2008) this was countered by several interviewees, one of whom thought they were the only ones who would appreciate the importance of the research:

Maybe they are targeting people in this position, I agree with that but I don’t think it’s exploitation, it’s just realisation that what you are doing may potentially help someone else ... I think people in this position are the right ones [to ask]. (M25: 1477–1508)

Another considered the alternatives:

No I don’t think it is [exploitative] ... [pause]. If you were that desperate to have a baby, yes, you’ll do anything. But ‘everything’ [anything] could go to the realms of going out and finding a man somewhere ... I’ve got a friend who said, ‘My husband’s ... you know, you and him’. Awww no! I said I’d rather it was clinical and not a turkey baster, thank you! ... medicine has to, and I don’t think exploitation is the right word for it, it’s got to seize its opportunities ... and they’re going to go through that treatment anyway so a few eggs from them ... for me, isn’t exploitation, it’s a medical opportunity. (M28: 1188–1231).

The importance of ‘doing good’ was in the background for some sharers anyway:

I don’t personally feel that I was taken advantage of at all ... I would’ve 100% done it happily, without any financial element involved ... so I didn’t feel that it was put on me and I felt I had to take it and like you say, exploited, because of it; I didn’t feel that at all. (M06: 1075–1099)

This suggests that the motivation to give eggs freely was always there and would have been offered even if the NESR had not existed. Another couple had similar feelings about wanting to help anyway – with the same proviso that they would do this once their treatment had been successful:

I’ve achieved what I want to achieve and have a lovely baby, if there’s anything at all I can do ... [NESR has] given more hope to women and families and there’s no exploitation there at all and if I’ve achieved what I want to achieve I would have no qualms, even if you contacted me in three years’ time and said, ‘we’re doing some research and would you be prepared to take part and it’s going to mean donating some more eggs’– great, I wouldn’t expect to get paid for it. (M27: 1819–45)

Of course, these are not the circumstances in which people are volunteering to give eggs. Their preference appears to be to provide eggs for research once they have their own baby – not while they are still trying to get that baby.

## Discussion

These data provide valuable insight into the views and experiences of women participating in one particular egg provision scheme. This is not the whole story of the NESR, as the range of analytic themes reminds us, let alone of egg provision more generally – but it is an important start.

To summarise briefly: concerns about exploitation are not uppermost in women’s accounts of their experiences of the NESR. Most grounds for acknowledging the potential of the NESR to be exploitative are dismissed and other grounds are cited for rejecting the charge outright. The grounds for acknowledging the exploitative potential of the NESR are insightful and echo some of the concerns raised in the literature. However, the charge is rejected as interviewees position the NESR within a landscape of fertility treatment where private fees are high, helping somebody else’s treatment has serious, lifelong, consequences and global markets are alienating and untrustworthy. In such a terrain the NESR seems a reasonable alternative. Interviewees did not enter the NESR naively but with a sense of what they regarded as acceptable and unacceptable. Those who rejected the charge of exploitation outright did so on the grounds that they had to take the initiative, there was no coercion, they felt well informed, they were gaining the chance of more treatment and they were helping research. Familiarity with, and trust in, the clinic and the regulated environment helped to support these views. Again there is similarity with the literature, where exploitation is conflated with other concerns, such as informed consent, but here that conflation is used as a counter to the charge, rather than a support for it.

It could be argued that interviewees are not debating exploitation but are simply rejecting criticisms of the NESR. However, since they do identify aspects that could be deemed exploitative and are not wholly uncritical of the scheme, offering suggestions for its reorganisation, this argument does not do justice to their utterances. Even if they were simply rejecting criticism that would suggest they think the positive aspects outweigh the negative, including any potential to be exploitative. It could also be argued that interviewees are simply reacting against suggestions that they personally had been exploited, as surely no one would want to admit to that. However, that also does a disservice to the interviewees’ complex and nuanced reasoning, including again the fact that they could see grounds for the charge. It also does not acknowledge the nature of the interviewer–interviewee relationships built up during an interview, the range of other (more sensitive) topics interviewees were willing to discuss, and the care with which this discussion was conducted.

It is important to consider these alternative interpretations but it is also important not to replace appropriate analytical scepticism too readily with a cynicism that ironicises interviewees’ discourses (Silverman 1985: 20–1). The interviewees’ reasoning on vulnerability and desperation, and on the potential losses and gains of the NESR, indicates that they are not merely passively responding to the irresistible carrot of reduced fees. They can, and do, change their minds about participation and they can, and do, decide the limits of what they will do in their attempts to have a baby, despite their desperation. Significantly, most choose not to share eggs for treatment even though this would give them an almost free IVF cycle. This counters concerns in the literature about undue inducement in the NESR undermining women’s capacity to make autonomous decisions.

However, there was a marked reluctance among IVF patients to provide eggs under the 12+ scheme and it is likely that interviewees in our project shared that reluctance. We have also seen that there is a very strong wish for more NHS-funded treatment, alongside suggestions for ways to reorganise the NESR such as providing eggs for research after successful treatment, rather than during treatment. Clinic staff noted that the local increase in NHS-funded IVF caused a decline in the number of volunteers for the NESR. Put together, these factors suggest that, although interviewees regard the decision to provide eggs under the NESR to be freely made, these are not necessarily decisions made under circumstances wholly of their own choosing. The fact that they do provide eggs under the NESR is because the fees discount makes it significantly different from the 12+, scheme, though it is notable that they highlight the increased chance of treatment that the reduced fees provide, rather than the discount itself. In this situation, eggs become seen as something that can be exchanged for that increased chance. The interviewees are similar to those women who declined to provide eggs under the 12+ scheme, as both groups see the eggs as very valuable in contributing to their goal of having a baby and therefore as too precious simply to give away. However, under the NESR, the eggs make a different sort of contribution, not used directly in treatment but used indirectly as a means of accessing more treatment. For those participating in the NESR eggs are still precious but their value lies in their status as an exchange commodity.

The gains and losses for the NESR volunteers are both material and symbolic. The material gains are the chance of extra treatment and, less obviously, gaining an alternative to the private/NHS/EST range of options for accessing treatment (not all of which are available to everyone). The symbolic gains lie in the sense of having more choice, someone providing extra help, having an extra chance of trying for a baby and of assisting research. It is important not to underestimate the value of these symbolic gains, given the practical and emotional difficulties of undergoing IVF (Rauprich *et al.* 2011). The losses should also be fully counted. While the material loss of eggs is accepted as part of the exchange, there is the lingering question of whether the very same egg that would have worked had been given away. A more embodied loss is the likely reduction in embryos available for freezing.

It is very clear that interviewees regard themselves as fully capable of making these evaluations of gains and losses, as well as deciding about alternatives, including not to go ahead. Nonetheless, several writers (Ballantyne 2008, Malmqvist 2011, Widdows 2009) warn, quite rightly, of the dangers of presuming that choice and autonomy resolve all concerns about exploitation even when individuals deny vehemently that they have been exploited, and even when those individuals benefit from the practice in question. Egg donation for stem cell research (though not the NESR) is one of the cases through which Widdows formulates her argument, highlighting the role of gender subordination in restricting women’s choices and increasing the possibilities for exploitation. She questions the usefulness of subjective definitions of exploitation, favouring objective calculations such as found in Marx. However, she also argues for the need to consider the ‘full social, political, economic and cultural context of egg provision for stem cell research’ (Widdows 2009: 13). We would argue that women’s own views and experiences are a vital part of that full context. Our data suggest that volunteers work within the duality of opportunity and constraint that the NESR provides within the broader context of inadequate NHS provision. Nonetheless, this does not mean that the NESR can simply be taken as a justification for normalising and extending egg provision elsewhere (Widdows 2009: 6). Lawlor (2011) reminds us that, even if one group is not exploited, consideration still needs to be given to protecting the interests of those like this group who might be in different circumstances. Egg provision for research is not one single practice: it takes many forms and the details of implementation of any particular scheme require careful scrutiny to avoid the actual and potential abuses already identified. The nuanced understandings that empirical investigations provide, of both subjective meanings and values and of structural constraints, are a vital part of that scrutiny.

One danger of relying on objective measures of exploitation is that debates can be reduced to following fairly crude understandings of Marxist surplus value theses and calculations of what levels of payment would constitute a fair return, what constitutes exploitative underpayment and what constitutes an undue inducement (Phillips 2011). Greater insight is offered by Waldby and Cooper’s analysis of the transition from gift to transactional economies in human tissues and of the need to understand the importance of women’s regenerative labour in stem cell industries. They point to the ways in which such developments involve ‘particular groups of women in complex negotiations over their role in bioeconomic activity’ (Waldby and Cooper 2010: 5). Our data provide subjective insights into the priorities of one group, especially their views on what counts as a fair return for their eggs, in their particular circumstances, with their particular goals. The data show their subjective management of the situation in which they find themselves.

Support for this approach comes from writers such as Wertheimer. We have seen in the egg provision literature the tendency to use exploitation as an umbrella term, conveying a forceful, but often undelineated, criticism of egg provision schemes. Wertheimer observes that ‘the concept of exploitation is typically invoked without much analysis or argument, as if its meaning and moral force were self-evident. They are not’ (Wertheimer 2008). He distinguishes between harmful exploitation (actions that harm another person by taking unfair advantage of them) and mutually advantageous exploitation, where one party is likely to gain more than the other, but both enter into the transaction because they both gain from it. He also distinguishes between non-consensual exploitation and consensual exploitation. Wertheimer argues that it is false to assume that a transaction is exploitative when offered to someone described as vulnerable or desperate, as long as the proposed transaction is reasonable. Clearly the views of those who are potentially exploitable, such as the NESR volunteers, need to be taken into account in determining what is reasonable and the value of what is gained or lost. Wertheimer’s work, while not the final word on exploitation, is useful for two reasons: (i) he demonstrates that exploitation is a contestable concept; ^6^ and (ii) he provides the potentially exploitable with agency, illustrating how important it is to take their evaluations into account.

If one combines the interviewees’ responses with the possibility of the creation of surplus value by the researchers (or, more likely, from any commercial advantage that derives from the research), the NESR might be seen as an example of mutually advantageous exploitation. However, what follows from this tentative conclusion? As Wertheimer says;

It is trivially true that it is wrong for A to gain from an action that unjustifiably harms or coerces B ... it is more difficult to explain when and why it might be wrong for A to gain from an action that benefits B and to which B voluntarily consents. (Wertheimer 2008)

Nor is it easy to decide whether ‘society’ should intervene in such cases. Wertheimer would argue that the state should not intervene in most cases of mutually advantageous exploitation. However, given the potential of egg provision to become more harmfully exploitative and given that, in the UK at least, SCNT research is publicly funded, there are grounds for robust state and professional oversight. Thompson (2007) argues that harm mitigation should be the central goal in protecting egg providers, which Waldby (2008) would combine with various industrial relation-type rights.

The suggestion that interviewees act towards their eggs in terms of their exchange value does, of course, edge towards the concerns about commodification. For some, the human body and its constituent parts should be excluded from such associations, as it is wrong to make ‘a saleable object of something that should be treated as having value in itself, irrespective of what use might be made of it’ (Widdows 2009: 18). One could ask from whence the authority of that ‘should’ comes (Haimes and Williams 2007). However, just as with embryos (Haimes *et al.* 2008) it appears, from our data at least, that eggs (and therefore perhaps other body parts too) have no single moral or cultural value or status but are viewed by different people in different ways and as having multiple values and meanings according to their location, context and time. For the interviewees, exchanging eggs for more treatment and therefore for a greater chance of having a baby is a reasonable thing to do. They are clear about their priorities and it is not easy for external commentators to arbitrate between the value (moral, cultural, economic) of eggs used as a means to an end and the value of couples’ wellbeing – let alone between the value of eggs and the increased possibility of a baby.

This suggests that interviewees can be seen as economic actors, getting what they deem to be a reasonable return for their regenerative labour, under the prevailing circumstances, exchanging or trading their eggs for the best deal available, as they see it, at the time. While they might not be acting quite as oocyte vendors in Waldby’s terms (2008) they are close to this. Indeed, these data add to understandings of how tissue economies operate in practice. There has been a failure, in some accounts of egg provision, to see women’s ability to evaluate the gains and losses for themselves, within a clear awareness of wider structural constraints and opportunities and then to act accordingly, in their own best interests.^7^ Just as there is no universal notion of the egg, there is no universal label that adequately describes egg provision: it will be valuable, exploitative, empowering or disrespectful, according to the conditions of provision, procurement, use and disposal and according to the end product being sought (stem cell lines/baby). It is the responsibility of social scientists to provide evidence of that variability so that women can be given genuine choices and so that best practices and worst offences can be acted upon. Probably the best protection derives from a full recognition of women’s vital (in all senses) role in underpinning developments in these aspects of stem cell research.

A focus on exploitation does not address all aspects of the NESR, just as studying the NESR does not address all the anxieties about egg provision worldwide. However, in providing empirical insights into both we have made an important start on furthering understandings of this challenging social practice. One of the important lessons to be learnt from this study is that it is not sufficient to label a practice exploitative or not: there are degrees and types of exploitation and non-exploitation, defined by different parties, in different ways, in different times and contexts. This is not an argument for avoiding discussing exploitation but the contrary: there is a need to engage with it, assiduously and conscientiously, to understand what it is, where and when it occurs, and why. The contribution that sociologists can make to these political, economic and moral debates about the definitions, manifestations and evaluations of exploitation is to provide evidence-based knowledge of the practices and transactions involved, teasing out the common factors that inform the creation of protections against exploitative practices or better still, to prevent exploitation altogether.

## Footnotes

1 We follow the established convention of using inverted commas to indicate when terms are highly contested. The term ‘share’ is used by the clinical researchers and, as will become clear throughout the article, is contested by others.

2 We use ‘provision’ rather than ‘donation’ since the latter can be laden with assumptions about ‘giving’, ‘gifting’ and ‘altruism’. Part of the goal of our research is to understand whether, how and why tissue is provided, so we cannot assume benign, or other, motivations.

3 Writers like Dickenson have been very effective in bringing ‘the lady’ back into consideration; consequently greater attention has now been paid to egg providers than to couples providing embryos for research (Haimes and Luce 2006).

4 ‘Volunteers’ is shorthand for ‘women and couples coming forward as potential egg “sharers” who might or might not have actually provided eggs’.

5 We have given all individuals a unique code to preserve their anonymity; we have also provided a reference to the line numbers in the transcript from which the quote is taken.

6 Lawlor (2011) suggests that concerns about exploitation are often better understood as concerns about inequality.

7 Furthermore, acting in their own interests does not eradicate altruistic beliefs and intentions; they simply act in ways that the current opportunities allow while noting the intention to act altruistically if such opportunities present themselves in the future.
